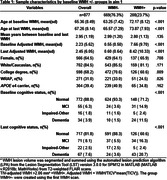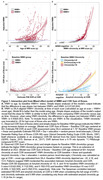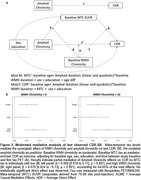# White matter hyperintensity onset, trajectories, and associations with cognitive decline in the presence of amyloid and tau

**DOI:** 10.1002/alz.093833

**Published:** 2025-01-09

**Authors:** Lianlian Du, Rebecca E. Langhough, Bruce P Hermann, Tobey J. Betthauser, Elizabeth M. Planalp, Erin M. Jonaitis, Ramiro Eduardo Rea Reyes, Leonardo A. Rivera‐Rivera, Karly Alex Cody, Nathaniel A. Chin, Robert V. Cadman, Kevin M. Johnson, Aaron S Field, Howard A. Rowley, Christian C. Pompa, Sarah C. Hudson, Brenda Ryther, Lia J. Sparks, Beryl Miess, Nia Norris, Celena M Ramsey, Kimberly D Mueller, Sanjay Asthana, Laura B. Eisenmenger, Bradley T. Christian, Sterling C. Johnson

**Affiliations:** ^1^ Department of Medicine, University of Wisconsin‐Madison School of Medicine and Public Health, Madison, WI USA; ^2^ Wisconsin Alzheimer’s Disease Research Center, Madison, WI USA; ^3^ Department of Neurology, University of Wisconsin‐Madison School of Medicine and Public Health, Madison, WI USA; ^4^ Wisconsin Alzheimer’s Institute, University of Wisconsin‐Madison School of Medicine and Public Health, Madison, WI USA; ^5^ Department of Medical Physics, University of Wisconsin‐Madison School of Medicine and Public Health, Madison, WI USA; ^6^ Department of Communication Sciences and Disorders, University of Wisconsin‐Madison, Madison, WI USA; ^7^ Wisconsin Alzheimer’s Disease Research Center, University of Wisconsin School of Medicine and Public Health, Madison, WI USA; ^8^ Department of Radiology, University of Wisconsin School of Medicine and Public Health, Madison, WI USA

## Abstract

**Background:**

Cognitive decline is often influenced by Alzheimer’s disease (AD) pathology (e.g., beta‐amyloid burden) and other pathology (e.g., vascular abnormalities). Amyloid onset and chronicity estimation using Sampled Iterative Local Approximation (SILA), enhanced our understanding of AD progression and its preclinical phase. This study has three aims: 1) estimate White Matter Hyperintensities (WMH) onset and chronicity; 2) assess whether baseline WMH chronicity/burden moderates the association between Aß chronicity/burden and Clinical Dementia Rating‐sum of boxes (CDR‐SB) trajectories; 3) explore whether tau burden mediates WMH and amyloid associations with CDR‐SB.

**Method:**

Participants were from the University of Wisconsin WRAP and ADRC cohorts and had completed at least one T1‐weighted and T2‐weighted FLAIR scans (Aim 1: n = 877, age 43‐93y). WMH values were aligned to a duration scale using SILA and WMH positivity threshold of ∼2.06 mL for WMH+ chronicity = 0. We compared mixed effects models with up to cubic time (age vs WMH chronicity) terms for characterizing WMH trajectories. We also examined baseline WMH burden*amyloid burden at each CDR‐SB assessment (PiB A+ DVR threshold ∼17CL; linear and quadratic amyloid burden) relative to longitudinal CDR‐SB (Aim 2; n = 426), and tau PET SUVR’s mediating effects on WMH*amyloid associations with last CDR‐SB (Aim 3; n = 385; meta‐temporal (MTC) SUVR; florquinitau tracer).

**Result:**

Mean(SD) age at baseline and last MRI were 65.36(8.49) and 67.26(8.14) years, respectively (sample details in Table 1). Comparisons of simple slopes (95%CI) show overlapping annualized WMH‐ slope estimates using age (0.028(0.022,0.034)) or chronicity (0.037(0.033,0.040)); in contrast, the WMH+ slopes are 2.26 times larger when aligned to WMH chronicity vs age (WMH+: 0.042(0.036,0.047), WMH‐: (0.095(0.091,0.10); Figure 1A&B). Whether biomarker “burden” was modeled as ±, estimated DVR, or chronicity (Figure 1C&D), significant interactions showed a synergistic effect of WMH and amyloid on accelerated CDR‐SB trajectory. Moderated mediation models revealed MTC‐tau accumulation partially mediated (∼65%) the synergistic effect of WMH and amyloid on last CDR‐SB (Figure 2).

**Conclusion:**

WMH accumulation follows a predictable trajectory post‐onset and appears to exacerbate cognitive decline in those with amyloid pathology. Future analyses will further elucidate the complex relationships between vascular risk, amyloid, tau accumulation and cognitive decline.